# Quality of and Recommendations for Relevant Clinical Practice Guidelines for COVID-19 Management: A Systematic Review and Critical Appraisal

**DOI:** 10.3389/fmed.2021.630765

**Published:** 2021-06-10

**Authors:** Yun-Yun Wang, Qiao Huang, Quan Shen, Hao Zi, Bing-Hui Li, Ming-Zhen Li, Shao-Hua He, Xian-Tao Zeng, Xiaomei Yao, Ying-Hui Jin

**Affiliations:** ^1^Center for Evidence-Based and Translational Medicine, Zhongnan Hospital of Wuhan University, Wuhan, China; ^2^Department of Evidence-Based Medicine and Clinical Epidemiology, Second Clinical College, Wuhan University, Wuhan, China; ^3^School of Health Sciences, Wuhan University, Wuhan, China; ^4^Precision Medicine Center, Second People's Hospital of Huaihua, Huaihua, China; ^5^Department of Health Research Methods, Evidence, and Impact, McMaster University, Hamilton, ON, Canada

**Keywords:** COVID-19, SARS-CoV-2, guideline, AGREE II, prophylaxis, diagnosis, treatments, discharge management

## Abstract

**Background:** The morbidity and mortality of coronavirus disease 2019 (COVID-19) are still increasing. This study aimed to assess the quality of relevant COVID-19 clinical practice guidelines (CPGs) and to compare the similarities and differences between recommendations.

**Methods:** A comprehensive search was conducted using electronic databases (PubMed, Embase, and Web of Science) and representative guidelines repositories from December 1, 2019, to August 11, 2020 (updated to April 5, 2021), to obtain eligible CPGs. The Appraisal of Guidelines for Research and Evaluation (AGREE II) tool was used to evaluate the quality of CPGs. Four authors extracted relevant information and completed data extraction forms. All data were analyzed using R version 3.6.0 software.

**Results:** In total, 39 CPGs were identified and the quality was not encouragingly high. The median score (interquartile range, IQR) of every domain from AGREE II for evidence-based CPGs (EB-CPGs) versus (vs.) consensus-based CPG (CB-CPGs) was 81.94% (75.00–84.72) vs. 58.33% (52.78–68.06) in scope and purpose, 59.72% (38.89–75.00) vs. 36.11% (33.33–36.11) in stakeholder involvement, 64.58% (32.29–71.88) vs. 22.92% (16.67–26.56) in rigor of development, 75.00% (52.78–86.81) vs. 52.78% (50.00–63.89) in clarity of presentation, 40.63% (22.40–62.50) vs. 20.83% (13.54–25.00) in applicability, and 58.33% (50.00–100.00) vs. 50.00% (50.00–77.08) in editorial independence, respectively. The methodological quality of EB-CPGs were significantly superior to the CB-CPGs in the majority of domains (*P* < 0.05). There was no agreement on diagnosis criteria of COVID-19. But a few guidelines show Remdesivir may be beneficial for the patients, hydroxychloroquine +/– azithromycin may not, and there were more consistent suggestions regarding discharge management. For instance, after discharge, isolation management and health status monitoring may be continued.

**Conclusions:** In general, the methodological quality of EB-CPGs is greater than CB-CPGs. However, it is still required to be further improved. Besides, the consistency of COVID-19 recommendations on topics such as diagnosis criteria is different. Of them, hydroxychloroquine +/– azithromycin may be not beneficial to treat patients with COVID-19, but remdesivir may be a favorable risk-benefit in severe COVID-19 infection; isolation management and health status monitoring after discharge may be still necessary. Chemoprophylaxis, including SARS-CoV 2 vaccines and antiviral drugs of COVID-19, still require more trials to confirm this.

## Introduction

The morbidity and mortality associated with coronavirus disease 2019 (COVID-19) are still increasing at present. According to the official website of World Health Organization (WHO), by 10 April 2021, there have been 134,308,070 confirmed cases of COVID-19, including 2,907,944 deaths worldwide (1). Containing the spread poses a challenge because of the rising number of infected people with high mortality and the highly contagious nature of COVID-19. Clinical practice guidelines (CPGs) have been defined as “statements that include recommendations intended to optimize patient care that are informed by a systematic review of evidence and a risk-benefit assessment of alternative care options” (2), and they play an important role in guiding clinical decisions about prevention, diagnosis, treatment and care. Some professional association, guideline development groups have issued successively COVID-19 management guidelines.

Previous reviews have also concentrated on methodological quality and recommendations for COVID-19 guidelines, but these have covered a narrow range of topics (3–6). The methods and reporting quality of practice guidelines for five different viruses causing public health emergencies of international concern, including the severe acute respiratory syndrome coronavirus 2, tended to be low, particularly in stakeholder involvement and applicability. There was also poor quality of recommendations for the use of antiviral drugs such as lopinavir-ritonavir, convalescent plasma, and intravenous immunoglobulins. Reverse transcription-polymerase chain reaction (RT-PCR) and Computed tomography (CT) were the most common diagnostic methods for COVID-19. Besides, there was no effective treatment against COVID-19; supportive therapy (mainly rest in bed, ensuring adequate calories, maintaining water-electrolyte balance, oxygen therapy, etc.) is the most significant treatment plan. Live evidence related to COVID-19 is still appearing on a daily basis, and live recommendations on chemoprophylaxis, diagnosis, and antiviral therapy are also being continuously updated. As for discharged patients, a small proportion of patients experienced reappearance of a positive test for SARS-CoV-2 during convalescence (7–9). As the number of cured patients increases, criteria for discharge management is also an important issue.

Thus, this review, based on a comprehensive literature search, has been conducted to compare the variations in recommendations within prophylaxis, diagnosis, antiviral treatment, and discharge management of COVID-19 and to assess their methodological quality. We aim to provide relatively more reliable suggestions for decision-making bodies regarding possible health problems to satisfy the needs of the public, providing guidance for government departments and COVID-19 prevention and control institutions.

## Methods

The review was performed according to preferred reporting items for systematic reviews and meta-analyses (PRISMA) guidelines (10).

### Search Strategy

We searched PubMed, Embase, and Web of Science. Additionally, eight representative guideline repositories were searched: World Health Organization (WHO), National Institute for Health and Care Excellence (NICE), Guidelines International Network (GIN), National Institutes of Health (NIH), Scottish Intercollegiate Guidelines Network (SIGN), Association of American Medical Colleges (AAMC), ECRI Guideline Trust, and Biochemical Genetic and Genomic (BIGG). A list of the websites with COVID-19 guidelines is presented in Supplementary Table 1. The search dates were from December 1, 2019, to August 11, 2020 (updated to April 5, 2021). The key words mainly included “severe acute respiratory syndrome coronavirus 2 or SARS-COV-2 or COVID-19 or COVID19 or 2019 coronavirus or 2019 novel coronavirus or 2019-nCoV or Novel coronavirus pneumonia or NCP or coronavirus disease-19 or coronavirus disease 2019” AND “guideline or guidance or recommendation or clinical practice guideline or consensus.” MeSH terms were used to search Title/Abstract. Furthermore, taking PubMed as an example, the retrieval strategy is shown in Supplementary Figure 1.

### Guidelines Identification

All guidelines related to COVID-19 published in English were included if they met the following criteria: (1) explicit recommendations on COVID-19 management (Which kind of agent can prevent COVID-19? Which strategy can be used to diagnose COVID-19 and identify and risk stratify patients with suspected or confirmed COVID-19? Which drugs can be used to treat patients with COVID-19? What are the discharge criteria for COVID-19, and what indicators are there for follow-up attention after discharge?); (2) evidence-based clinical practice guidelines (EB-CPGs) or consensus-based guidelines (CB-CPGs); and (3) updated versions of CPGs if multiple versions of the guidelines exists. To determine the eligible guidelines, EB-CPGs were defined as having recommendations based on a systematic literature search and literature quality assessment or grade for evidence and recommendation; CB-CPGs were defined as having recommendations developed by multidisciplinary experts (such as frontline clinicians) based on their experience or the existing literature using a consensus method rather than a systematic review.

We excluded (1) translated versions, interpretations, and summaries of existing CPGs; (2) regional or hospital protocols for COVID-19; and (3) CPGs without full text access.

### Data Extraction

Four reviewers independently extracted the details of the guidelines relevant to their characteristics using a standardized data collection form. Extracted data included guideline title, date of publication, publication country/region, guideline developers, target population, development method (evidence-based or consensus-based), topic, funding, and the related recommendations. Another reviewer examined the data extraction forms to make sure no errors had occurred. Disagreements were resolved by consensus.

### Methodological Quality Appraisal

Two reviewers independently evaluated the quality of each included guideline using the widely accepted CPG assessment tool—AGREE II, which is composed of 23 items within 6 domains including “scope and purpose,” “stakeholder involvement,” “rigor of development,” “clarity of presentation,” “applicability,” and “editorial independence” (11, 12). Details of each domain are shown in Supplementary Table 2. Each item was scored from 1 (strongly disagree) to 7 (strongly agree). We calculated each domain score for every eligible CPG individually using the following formula provided by the AGREE II tool: (obtained score–minimal possible score)/(maximal possible score–minimal possible score) × 100% (11).

### Guideline Recommendations Synthesis

We performed a textual descriptive synthesis to analyze eligible CPGs from four aspects: chemoprophylaxis; diagnosis; antiviral therapy; and discharge management.

### Statistical Analysis

Descriptive statistical analyses were performed. Data for each AGREE II domain of every included CPG were presented as medians and interquartile ranges (IQRs). The scores of EB-CPGs and CB-CPGs in each domain were compared using Wilcoxon Rank-Sum Test. A *P* < 0.05 was regarded as significance. Intraclass correlation coefficients (ICCs) with a 95% confidence interval (CI) were calculated to evaluate the agreement among two assessors for each domain. The degree of agreement between 0.00 and 0.40 was considered poor, 0.41 to 0.75 was good, and 0.75 to 1.00 was excellent (13). All the data were analyzed using R version 3.6.0 software (The R Foundation for Statistical Computing, Vienna, Austria) for Windows.

## Results

### Guidelines Identification and Selection

**Figure 1:**
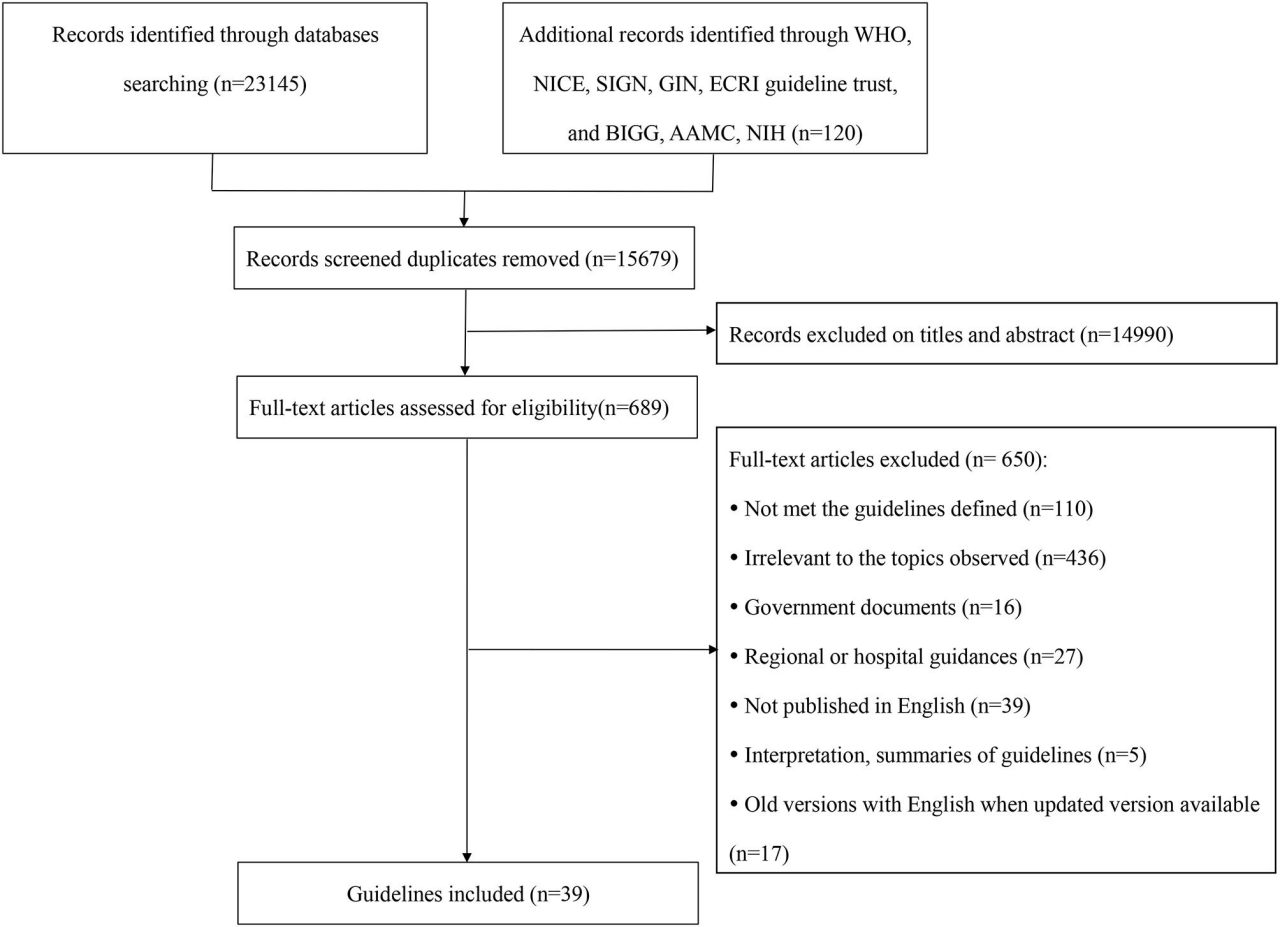
Flow chart of guidelines identification and selection.

Figure 1 presents the flow chart of guidelines identification, and 39 CPGs were eventually included (14–52).

### Characteristics of Included Guidelines

**Figure 2:**
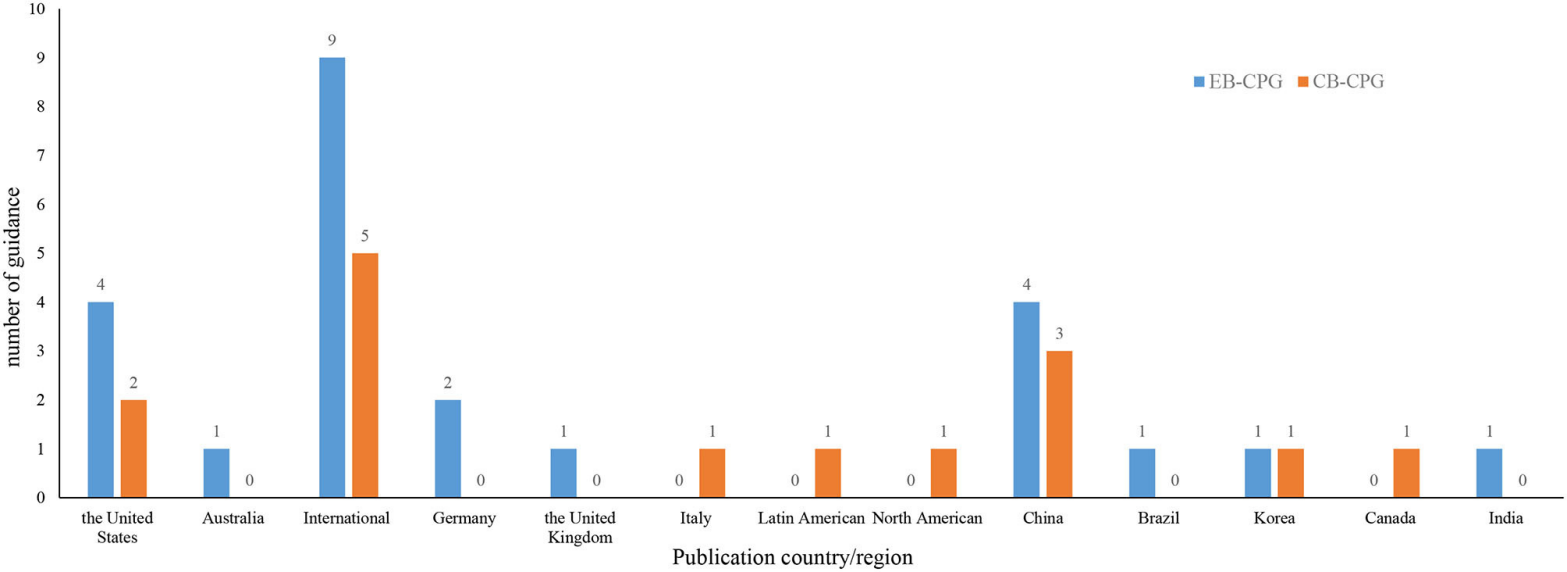
Distribution of publication country/region in the guidelines included.

As Supplementary Table 3 shows, the guidelines were published from February 6, 2020, to April 5, 2021. Of them, 15 guidelines were CB-CPGs and 24 were EB-CPGs, and 15 received funding support. Among the recommendations in these 39 CPGs, 8 were on chemoprophylaxis, 18 on diagnosis, 1 on identification and triage of patients with COVID-19, 25 on antiviral drugs, and 6 on discharge. The guidelines were mainly developed by the United States, China, or other international organization or cooperation (See Figure 2).

### Guidelines' Quality

**Figure 3:**
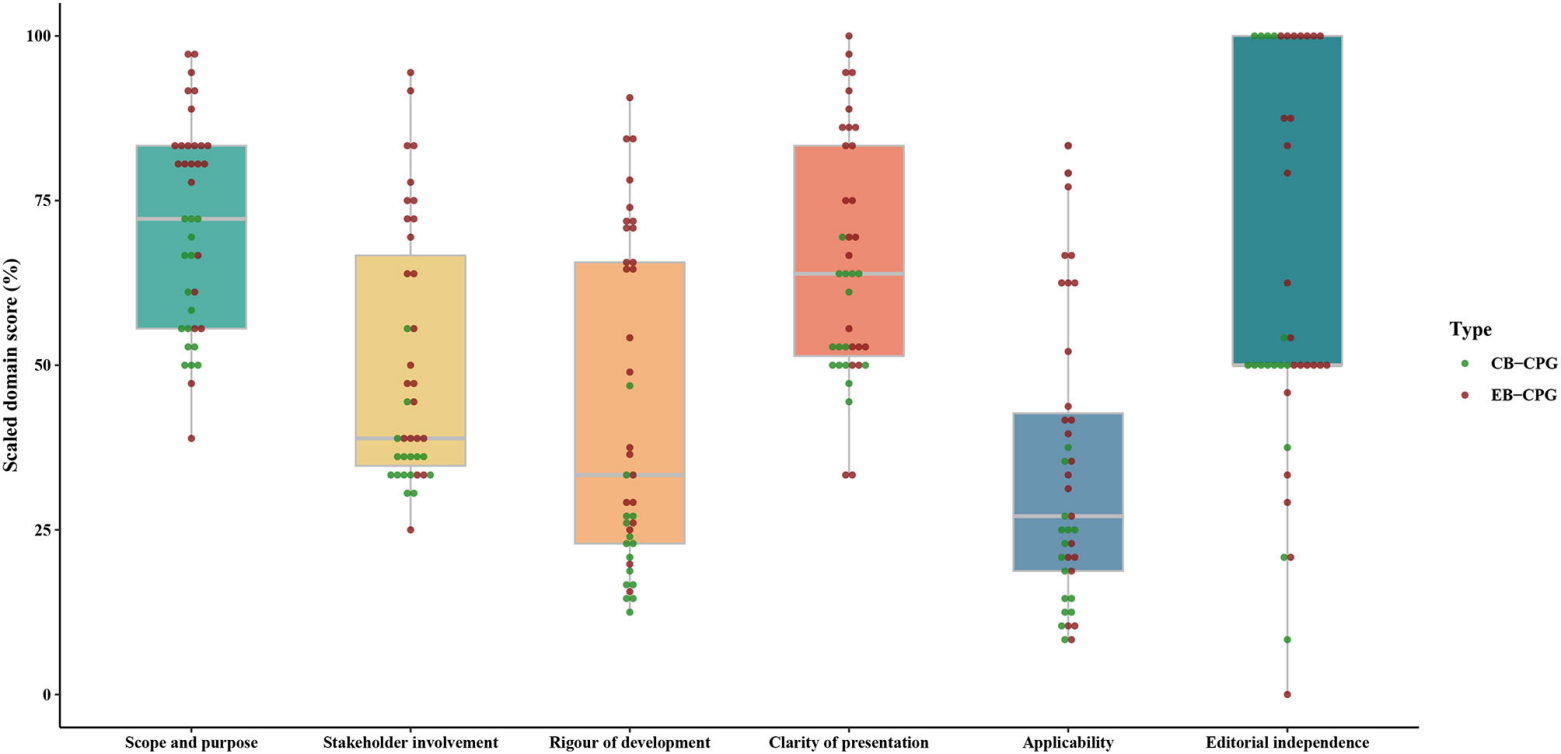
The summary of scores for each domain over all included guidelines.

The ICC values for all six domains of AGREE II were over 0.75, indicating a high consistency on the scores between the two assessors. As shown in Supplementary Table 4, Table 1, Figure 3, the final domain score of every guideline across all domains ranged from 0% (Domain 6 of editorial independence in 1 guidelines) (51) to 100% (Domain 6 in 11 guidelines) (16, 19, 25, 29, 31, 32, 34, 44, 46–48). Regarding the score of each domain across all guidelines, for EB-CPGs, the score of Domain 5 (applicability) was the lowest with a median score of 40.63% (IQR 22.40–62.50), the median scores of Domains 1, 2, 3, 4, 6 (scope and purpose, stakeholder involvement, rigor of development, clarity of presentation, editorial independence) were 81.94% (IQR 75.00–84.72), 59.72% (IQR 38.89–75.00), 64.58% (IQR 32.29–71.88), 75.00% (IQR 52.78–86.81), and 58.33% (IQR 50.00–100.00), respectively. For CB-CPGs, Domain 1 scored highest with a median score of 58.33% (IQR 52.78–68.06), Domain 5 scored lowest with median scores of 20.83% (13.54–25.00), and the median scores of Domains 2, 3, 4, 6 were 36.11% (33.33–36.11), 22.92% (16.67–26.56), 52.78% (50.00–63.89), and 50.00% (50.00–77.08), respectively. In addition, EB-CPGs were significantly superior to the CB-CPGs in the domain 1, 2, 3, 4, 5(*P* < 0.05).

**Table 1:** The difference of quality between EB-CPGs and CB-CPGs.

**Domains**	**Scope and purpose**	**Stakeholder involvement**	**Rigor of development**	**Clarity of presentation**	**Applicability**	**Editorial independence**
*Z*	−3.493	−3.744	−4.102	−2.828	−2.905	−0.714
*P*	0.000	0.000	0.000	0.005	0.004	0.475

### Synthesis of Recommendations

Five EB-CPGs (15, 19, 20, 24, 34) and three CB-CPGs (17, 27, 41) focused on the chemoprophylaxis of COVID-19. Two EB-CPGs of them recommended not to use hydroxychloroquine for COVID-19 pre-exposure prophylaxis or post-exposure prophylaxis outside the setting of a clinical trial (15, 19); two EB-CPG (20, 24) and two CB-CPGs (17, 27) recommended SARS-CoV vaccine for COVID-19 prevention; one CB-CPG (41) suggested that a few traditional Chinese medicine may be beneficial for COVID-19 prevention, for example, Youngyopaedoc-san plus Bojungikgitang, Youngyopaedoc-san plus Saengmaek-san (See Table 2).

**Table 2:** Recommendations on chemoprophylaxis of COVID-19.

**Guidelines title**	**Drugs**	**Pre-exposure prophylaxis**	**Post-exposure prophylaxis**
**EB-CPG**			
Australian guidelines for the clinical care of people with COVID-19 (15)	Hydroxychloroquine	*	**
WHO living guideline: drugs to prevent COVID-19 (19)	Hydroxychloroquine	***	***
Coronavirus disease 2019 (COVID-19) treatment guidelines (20)	Any drugs	****	*****
	Vaccine	***	***
2021 update of the AGIHO guideline on evidence-based management of COVID-19 in patients with cancer regarding diagnostics, viral shedding, vaccination and therapy (24)	Vaccine		
Chemoprophylaxis, diagnosis, treatments, and discharge management of COVID-19: an evidence-based clinical practice guideline (updated version) (34)	Any drugs		
**CB-CPG**			
American College of Rheumatology guidance for COVID-19 vaccination in patients with rheumatic and musculoskeletal diseases–Version 1 (17)	Vaccine	***	***
SARS-CoV-2 vaccination for patients with inflammatory bowel diseases: recommendations from an international consensus meeting (27)	Vaccine	******	
A consensus guideline of herbal medicine for coronavirus disease 2019 (41)	Youngyopaedoc-san + Bojungikgitang (Lianqiao baidu san + Buzhong Yiqi Tang)		
	Youngyopaedoc-san + Saengmaek-san (Lianqiao baidu san + Shengmai Yin)		
	Youngyopaedoc-san + Bulhwangeumjeonggi-san (Lianqiao baidu san + Buhuanjin Zhengqi San)		
	Youngyopaedoc-san + Bojungikgi-tang (Lianqiao baidu san + Buzhong Yiqi Tang)		
			Recommended
			Not recommended
			Not reported
			Insufficient evidence to recommend or not recommend

*For healthcare workers with no active COVID-19, do not use hydroxychloroquine for pre-exposure prophylaxis outside of randomized trials with appropriate ethical approval; **For people exposed to individuals with severe acute respiratory syndrome coronavirus 2 infection, do not use hydroxychloroquine for post-exposure prophylaxis outside of randomized trials with appropriate ethical approval; ***No specific indication of pre-exposure or post-exposure prophylaxis; ****Recommending against the use of any drugs for severe acute respiratory syndrome coronavirus 2 pre-exposure prophylaxis, except in a clinical trial; *****Recommending against the use of hydroxychloroquine for SARS-CoV-2 post-exposure prophylaxis,against the use of other drugs for SARS-CoV-2 post-exposure prophylaxis, except in a clinical trial; ******Patients with inflammatory bowel diseases who are receiving immune-modifying therapies should not receive live virus vaccines.

*EB-CPGs, Evidence-based clinical practice guidelines; CB-CPGs, Consensus-based guidelines*.

In total, 11 EB-CPGs (14, 20, 21, 24, 26, 30, 34, 36, 42, 43, 51) and 7 CB-CPGs (32, 33, 44, 46, 48, 49, 52) reported the diagnostic criteria for COVID-19 (See Table 3). The diagnosis of SARS-CoV-2 infection was mainly based on RT-PCR test, serum-specific antibodies IgM and IgG test, epidemiological history, and clinical manifestations in one EB-CPG (34) and one CB-CPG (46). However, nine EB-CPGs (14, 20, 21, 24, 26, 30, 36, 43, 51) and six CB-CPGs (32, 33, 44, 48, 49, 52) only focus on one or two of the above criteria. Three CPGs (two EB-CPGs, one CB-CPG) (20, 26, 32) did not suggest SARS-CoV-2 antibody tests for diagnosis of current infection with COVID-19 or as the sole basis or to routinely to diagnose active COVID-19 in symptomatic pregnant women with negative RT-PCR. Two CPGs (one EB-CPG, one CB-CPG) (32, 42) did not recommend that CT scan were used routinely in the diagnosis of COVID-19 in children or symptomatic pregnant women. In addition, one EB-CPG (31) provided some suggestions on how to predict whether a patient is COVID-19 positive, validated triage and severity of illness, risk stratify patients with suspected or confirmed COVID-19 in low- and middle-income countries.

**Table 3:** Recommendations on diagnosis criteria of COVID-19.

**Guidelines title**	**Etiological criteria**	**Serological criteria**	**Epidemiological history and clinical manifestations**	**CXR or chest CT**
**EB-CPG**				
IDSA guidelines on the treatment and management of patients with COVID-19 (14)		*		
Coronavirus disease 2019 (COVID-19) treatment guidelines (20)		**		
Surviving Sepsis Campaign guidelines on the management of adults with coronavirus disease 2019 (COVID-19) in the ICU: first update (21)				
2021 update of the AGIHO guideline on evidence-based management of COVID-19 in patients with cancer regarding diagnostics, viral shedding, vaccination, and therapy (24)				
Clinical management of COVID-19 patients: living guidance (26)		***		
Clinical practice guideline: recommendations on inpatient treatment of patients with COVID-19 (30)				
Chemoprophylaxis, diagnosis, treatments, and discharge management of COVID-19: an evidence-based clinical practice guideline (updated version) (34)				
Use of chest imaging in the diagnosis and management of COVID-19: a WHO rapid advice guide (36)				****
Rapid advice guidelines for management of children with COVID-19 (42)				*****
Expert consensus for managing pregnant women and neonates born to mothers with suspected or confirmed novel coronavirus (COVID-19) infection (43)				******
Perinatal-neonatal management of COVID-19 infection (51)				
**CB-CPG**				
Clinical management of coronavirus disease 2019 (COVID-19) in pregnancy: recommendations of WAPM-World association of perinatal medicine (32)		*******		********
Algorithms for testing COVID-19 focused on use of RT-PCR and high-affinity serological testing: a consensus statement from apanel of Latin American experts (33)				
Canadian society of thoracic radiology/Canadian association of radiologists consensus statement regarding chest imaging in suspected and confirmed COVID-19 (44)				
Updated diagnosis, treatment and prevention of COVID-19 in children: experts' consensus statement (condensed version of the second edition) (46)				
Imaging of coronavirus disease 2019: a Chinese expert consensus statement (48)				
The role of chest imaging in patient management during the COVID-19 pandemic (49)				
Chinese expert consensus on the perinatal and neonatal management for the prevention and control of the 2019 novel coronavirus infection (first edition) (52)				
				Recommended
				Not recommended
				Not reported

*When SARS-CoV-2 infection requires laboratory confirmation for clinical or epidemiological purposes, testing for SARS-CoV-2 IgG or total antibody 3 to 4 weeks after symptom onset to detect evidence of past SARS-CoV-2 infection;using IgG antibody to provide evidence of COVID-19 infection in symptomatic patients with a high clinical suspicion and repeatedly negative NAAT testing; In pediatric patients with multisystem inflammatory syndrome, using both IgG antibody and NAAT to provide evidence of current or past COVID-19 infection; **Not recommended as the sole basis; ***SARS-CoV-2 antibody tests are not recommended for diagnosis of current infection with COVID-19; ****For symptomatic patients with suspected COVID-19, using chest imaging for the diagnostic workup of COVID-19 when RT-PCR testing is not available; RT-PCR testing is available but results are delayed; and initial RT-PCR testing is negative but with high clinical of suspicion of COVID-19; *****CT scan should not be used routinely in the diagnosis of COVID-19 in children; ******Pregnant women with suspected COVID-19 infection; *******Not recommend routine serological testing to diagnose active COVID-19 in symptomatic pregnant women with negative RT-PCR; ********Not currently recommend using chest CT scans or X-rays as a first-line test for diagnosing COVID-19 in symptomatic pregnant women.

*EB-CPG, Evidence-based guideline; CB-CPG, Consensus-based guideline; CXR, chest radiography; chest CT, chest computed tomography. Etiological criteria: testing positive for SARS-CoV-2 by real-time polymerase chain reaction (PCR) and highly homologous genetic sequencing of respiratory tract or blood samples with the known SARS-CoV-2; Serological criteria: positive results of serum-specific antibodies IgM and IgG test, specifying serum-specific antibody IgG changed from negative to positive or increased four-fold or higher from that in the acute phase during the recovery period; Epidemiological history: involved noting whether the patients had a travel or residence history in a community with infected cases reported in China or a country or region with a serious epidemic, a history of contacting patients infected with SARS-Cov-2, a history of contacting patients with fever or respiratory symptoms from communities with reported cases in China or countries or regions with serious epidemics, clustered cases within 14 days prior to disease onset. Clinical manifestations: mainly consisted of fever, fatigue, dry cough, and/or other respiratory symptoms; COVID 19 imaging features and, in the early stage of the disease, the total number of leukocytes was normal or decreased, and the lymphocyte count was decreased*.

In total, 18 EB-CPGs (14–16, 18, 20, 21, 24–26, 34, 37, 39, 40, 42, 45, 47, 50, 51) and 7 CB-CPGs (22, 23, 28, 32, 38, 41, 46) provided suggestions on antivirals treatment for COVID-19. As shown in Table 4, there were no consistent views on effective and validated antiviral drugs such as hydroxychloroquine/chloroquine plus azithromycin, lopinavir/ritonavir, convalescent plasma for the treatment in clinical scenarios. The majority of guidelines agreed that some antiviral drugs such as Remdesivir can be used in the context of clinical trials or under special conditions such as severe and critical patients. Two EB-CPGs (34, 39) and one CB-CPG (41) provided a traditional Chinese medicine treatment plan for COVID-19.

**Table 4:** Recommendations on antivirals drugs for COVID-19.

**Guidelines title**	**Hydroxychloroquine +/– azithromycin**	**Lopinavir/ritonavir**	**Corticosteroids**	**Tocilizumab**	**Convalescent plasma**	**Remdesivir**	**Antibiotics**	**Famotidine**	**Bamlanivimab+/– etesevimab**	**Azithromycin**	**Baloxavir marboxil**	**Chloroquine**	**Favipiravir**	**Recombinant human granulocyte colony-stimulating factor**	**Sarilumab**	**Umifenovir**	**Interferon alfa**	**Interferon beta**	**Immunoglobulins**	**Traditional Chinese Medicine**
**EB-CPG**																				
IDSA guidelines on the treatment and management of patients with COVID-19 (14)			*	**	***	****		*****	******											
Australian guidelines for the clinical care of people with COVID-19 (15)			*** ****	**** ****		**** *** **			**** *** ***		**** **** **	*** *** ****	*** **** ***	**** **** **	*** **** ***	**** *** ***				
COVID-19 rapid guideline: managing COVID-19 (16)			**** **** ***	**** **** ****		**** **** *****									**** ****** ****					
Management of hospitalized adults with coronavirus disease-19 (COVID-19): a European Respiratory Society living guideline (18)			**** ****** *****																	
Coronavirus disease 2019 (COVID-19) treatment guidelines (20)			***** **** **** ***			***** **** **** ***														
Surviving Sepsis Campaign guidelines on the management of adults with coronavirus disease 2019 (COVID-19) in the ICU: first update (21)			**** ******* ******		****** ***** *******	***** **** **** ******													***** ***** ***** *****	
2021 update of the AGIHO guideline on evidence-based management of COVID-19 in patients with cancer regarding diagnostics, viral shedding, vaccination and therapy (24)																				
Should remdesivir be used for the treatment of patients with COVID-19? rapid, living practice points from the American College of Physicians (version 2) (25)						***** **** ***** *******														
Clinical management of COVID-19 patients: living guidance (26)	**** **** ***** **** *****	*** **** ***** ****** ****	**** **** **** **** *** ***	**** **** ***** ***** ****	**** ***** **** **** *****	**** **** ***** ***** ****	**** ***** **** *** ******	*** *** ***** ***** *** ***	**** **** **** **** ******	**** **** **** ***** *****	**** **** ***** ***** ****	**** **** ***** ***** ****	***** ***** ****** ******	**** ****** ****** ******	*** ***** ***** **** *****	**** ***** ****** **** ***	**** ***** ***** ***** ***	***** **** ***** **** ****	***** **** **** ****** ***	
Chemoprophylaxis, diagnosis, treatments, and discharge management of COVID-19: an evidence-based clinical practice guideline (updated version) (34)	***** ****** ***** **** ***																			
Remdesivir for severe covid-19: a clinical practice guideline (37)						****** ***** ***** **** ****														
Traditional Chinese medicine guidelines for coronavirus disease 2019 (39)																				
Guidelines for the pharmacological treatment of COVID-19 (40)							***** **** ****** ***** *****													
Rapid advice guidelines for management of children with COVID-19 (42)		**** ****** ***** **** ***	**** ***** ***** **** ****			***** *** ***** ***** ****	**** **** ****** ***** *** ***										****** **** **** **** ****	*** **** **** ***** ******	****** ***** ***** ****	
Treatment of patients with non-severe and severe coronavirus disease 2019: an evidence based guideline (45)			***** **** ***** **** **** ****		**** **** ***** **** **** **** **															
Interim guidelines on antiviral therapy for COVID-19 (47)						***							***							
Guideline for critical care of seriously ill adults patients with coronavirus (COVID-19) in the Americans (50)			****** ***** ***** **** **** ****				**** **** **** ***** ***** **** ***													
Perinatal-neonatal management of COVID-19 infection (51)		****** ***** ****** ***** ***** ***																		
**CB-CPG**																				
COVID-19 convalescent plasma: interim recommendations from the AABB (22)																				
Multicenter interim guidance on use of antivirals for children with coronavirus disease 2019/severe acute respiratory syndrome coronavirus 2 (23)						***** ***** ****** ***** ***** *****														
Therapeutic strategies for severe COVID-19: a position paper from the Italian Society of Infectious and Tropical Diseases (SIMIT) (28)						***** ***** ****** ****** **********	***** ****** ****** ***** ***													
Clinical management of coronavirus disease 2019 (COVID-19) in pregnancy: recommendations of WAPM-World Association of Perinatal Medicine (32)				***** ***** ***** **** *** ***********		** **** **** ***** ***** ****							***** ***** **** ****** ****** ***** ***							
Updated guidance on the management of COVID-19: from an American thoracic society/European respiratory society coordinated international task force (38)																				
A consensus guideline of herbal medicine for coronavirus disease 2019 (41)																				
Updated diagnosis, treatment and prevention of COVID-19 in children: experts' consensus statement (condensed version of the second edition) (46)							***** ***** ***** ***** *****												***** ******* ***** **** ***	
																				Recommended
																				Not recommended
																				Not reported
																				Insufficient evidence to recommend or not recommend

*Among hospitalized severe or critically ill patients with COVID-19; **Among hospitalized adults with progressive severe or critical COVID-19 who have elevated markers of systemic inflammation; ***Only in the context of a clinical trial; ****In hospitalized patients with severe COVID-19; *****Hospitalized patients with severe COVID-19, not using famotidine use for the sole purpose of treating COVID-19 outside of the context of a clinical trial; ******Ambulatory patients with mild to moderate COVID-19 at high risk for progression to severe disease; *******Adults with COVID-19 or pregnant or breastfeeding women with COVID-19 or children and adolescents with acute COVID-19 who are receiving oxygen (including mechanically ventilated patients); ********Adults or children and adolescents who require supplemental oxygen; *********Adults or pregnant or breastfeeding women hospitalized with moderate to severe COVID-19 who do not require ventilation; **********Not use outside of the context of a clinical trial; ***********People with COVID-19 who: need supplemental oxygen to meet their prescribed oxygen saturation levels or have a level of hypoxia that needs supplemental oxygen but who are unable to have or tolerate it; ************Adults in hospital with COVID-19 if all of the following apply: having or have completed a course of corticosteroids such as dexamethasone, unless they cannot have corticosteroids they have not had another interleukin-6 inhibitor during this admission there is no evidence of a bacterial or viral infection (other than SARS-CoV-2) that might be worsened by tocilizumab. And they either: need supplemental oxygen and have a C-reactive protein level of 75 mg/l or more, or are within 48 h of starting high-flow nasal oxygen, continuous positive airway pressure, non-invasive ventilation or invasive mechanical ventilation; *************COVID-19 pneumonia in adults, and young people 12 years and over weighing 40 kg or more, who are in hospital and on supplemental oxygen but not on invasive mechanical ventilation; **************Adults in hospital with COVID-19 only if tocilizumab cannot be used or is unavailable. Use the same eligibility criteria as those for tocilizumab. That is, if all of the following apply: they are having or have completed a course of corticosteroids such as dexamethasone, unless they cannot have corticosteroids they have not had another interleukin-6 inhibitor during this admission there is no evidence of a bacterial or viral infection (other than SARS-CoV-2) that might be worsened by sarilumab. And they either need supplemental oxygen and have a C-reactive protein level of 75 mg/l or more or are within 48 h of starting high-flow nasal oxygen, continuous positive airway pressure, non-invasive ventilation or invasive mechanical ventilation; ***************Patients with COVID-19 requiring oxygen, non-invasive ventilation or invasive mechanical ventilation; ****************Hospitalized but requires supplemental oxygen; *****************For adults with severe or critical COVID-19; ******************For adults with severe or critical COVID-19 outside clinical trials; *******************For adults with severe COVID-19 who do not require mechanical ventilation; ********************In critically ill adults with COVID-19 or Children; *********************Hospitalized patients with COVID-19 who do not require mechanical ventilation or ECMO or hospitalized patients with COVID-19 who require mechanical ventilation or ECMO within a 5-day course; **********************Not using unproven drugs not be administered as treatment or prophylaxis for COVID-19, outside of the context of clinical trials; ***********************Not using the combination of HCQ and azithromycin; ************************In severe covid-19; *************************COVID-19 patients with suspected bacterial coinfection; **************************Patients with severe coronavirus disease 2019 and acute respiratory distress syndrome; ***************************Not using convalescent plasma in patients with severe COVID-19; ****************************In mechanically ventilated adults with COVID-19 and respiratory failure (without ARDS), suggesting against the use of systemic corticosteroids; *****************************The administration of antibiotics should be initiated within an hour of assessing the patient. Antibiotic therapy should be deescalated on the basis of microbiological results and clinical judgment; ******************************If any of the following criteria are met: hypoxia, hypotension, new onset organ dysfunction (one or more of Increase in creatinine by 50% from baseline, GFR reduction by >25% from baseline or urine output of <0.5 ml/kg for 6 h), Reduction of GCS by 2 or more, or Any other organ dysfunction; *******************************Only in children with positive SARS-CoV-2 viral testing; used only within the context of a clinical trial in outpatients and hospitalized patients with asymptomatic, mild, or moderate COVID; suggested for children with severe COVID-19; ********************************Oxygen support only no mechanical ventilation; *********************************Tocilizumab may be considered for off-label use in pregnant women who have severe or critical COVID-19 with the suspicion of cytokine activation syndrome with elevated IL-6 levels as a last resort or based on a clinical research protocol; **********************************In pregnancy.

*EB-CPG, Evidence-based guideline; CB-CPG, Consensus-based guideline*.

As presented in Tables 5A,B, four EB-CPGs (29, 34, 36, 51) and two CB-CPGs (35, 52) concentrated on the discharge management of COVID-19. The criteria were mainly based on temperature returning to normal more than 3 days, improvement in respiratory symptoms and negative results from two successive nucleic acid test of respiratory samples (with a sampling interval of at least 1 day). Besides, three EB-CPGs (29, 34, 51) and one CB-CPG (35) described the relevant precautions after discharge. For example, isolation management should be continued, and the patients should wear a mask if necessary.

**Table 5A:** Recommendations on discharge criteria of COVID-19.

**Guidelines title**	**Body temperature**	**Respiratory symptoms**	**Pulmonary imaging**	**Detection of SARS-CoV-2 nucleic acid**
**EB-CPG**				
Pragmatic recommendations for tracheostomy, discharge, and rehabilitation measures in hospitalized patients recovering from severe COVID-19 in low- and middle-income countries (29)	*	**		
Chemoprophylaxis, diagnosis, treatments, and discharge management of COVID-19: an evidence-based clinical practice guideline (updated version) (34)	***	**	****	*****
Use of chest imaging in the diagnosis and management of COVID-19: a WHO rapid advice guide (36)			******	
**CB-CPG**				
Chinese expert consensus on the perinatal and neonatal management for the prevention and control of the 2019 novel coronavirus infection (first edition) (52)	***	**	****	*****
				Recommended
				Not recommended
				Not reported

*Afebrile for ≥24 h; **Substantially improved respiratory symptoms; ***Temperature returned to normal for more than 3 days; ****Significant absorption of pulmonary chest lesions; *****Two consecutive negative nucleic acid tests from sputum, nasopharyngeal swabs, or other respiratory tract samples (at least 24 h between samples); ******For hospitalized patients with COVID-19 whose symptoms are resolved, not using chest imaging in addition to clinical and/or laboratory assessment to inform the decision regarding discharge.

*EB-CPG, Evidence-based guideline; CB-CPG, Consensus-based guideline*.

**Table 5B:** Recommendations on precautions after discharge of COVID-19.

**Guidelines title**	**Isolation management**	**Health examination**	**Personal prevention**	**Points for attention**
**EB-CPG**				
Pragmatic recommendations for tracheostomy, discharge, and rehabilitation measures in hospitalized patients recovering from severe COVID-19 in low- and middle-income countries (29)	*		**	***
Chemoprophylaxis, diagnosis, treatments, and discharge management of COVID-19: an evidence-based clinical practice guideline (updated version) (34)	****	*****		Not reported
Perinatal-neonatal management of COVID-19 infection (51)		******	*******	
**CB-CPG**				
COVID-19: interim guidance on rehabilitation in the hospital and post-hospital phase from a European respiratory society- and American thoracic society-coordinated international task force (35)		********		*********
				Recommended
				Not reported

*Following local/regional/national deisolation, or ability to self-isolate adequately for a minimum of 10 days following the onset of symptoms, if applicable; **All patients and caregivers receive comprehensive education on adequate hygiene and the importance of mask-wearing, including for close contacts; ***Taking into consideration the capability of primary caregivers to provide the necessary care to meet the psychological, physical, and neurocognitive needs; ****Discharged patients may be quarantined for 2 weeks; *****PCR tests can be performed at 2 and 4 weeks after discharge; ******Early discharge to home may be followed by a telephonic follow-up or home visit by a designated nurse; *******Mothers should practice respiratory hygiene and wear a mask while breastfeeding and providing other care to the baby; they should routinely clean and disinfect all the surfaces; ********At 6–8 weeks following discharge, a formal assessment of physical and emotional functioning for patients with COVID-19; a formal psychological assessment for COVID-19 survivors with symptoms of psychological distress; *********At 6–8 weeks following discharge, doing regular daily activities in the first 6–8 weeks after hospital discharge; nutritional support for COVID-19 survivors with loss of lower-limb muscle mass, a musclestrengthening programme for COVID-19 survivors with loss of lower-limb muscle mass and/or function; a comprehensive pulmonary rehabilitation programme for COVID-19 survivors with pre-existing/ongoing lung function impairment; a comprehensive rehabilitation programme for COVID-19 survivors with a need for rehabilitative interventions.

*EB-CPG, Evidence-based guideline; CB-CPG, Consensus-based guideline*.

## Discussion

EB-CPGs and CB-CPGs play an important role in this pandemic, which is constantly being updated. The first EB-CPGs were published on Feb 6, 2020 (53); the first protocol of the updated EB-CPG was released on March 7, 2020 (54). Finally, 39 CPGs were included in this review. The methodological quality of EB-CPGs is better than CB-CPGs because the median score with IQR is statistically significantly higher in EB-CPGs for domains of the AGREE II assessment tool in general. However, they all still need to be further improved, especially in the areas of gathering and synthesizing reliable the latest up-to-date information, involving the target population in guideline development and improving the implementability of the recommendations. Recommendations relevant to chemoprophylaxis, diagnosis, antiviral drugs, and discharge management of COVID-19 showed small differences.

COVID-19 is a newly identified infectious disease, which poses a significant threat to both the general population and health care workers. In the early stage of the pandemic, the absolute lack of direct evidence is the biggest challenge for guideline development. A large number of CB-CPGs and EB-CPGs in accordance with experience of frontline health professionals, such as experts in infectious disease, medical imaging, and clinical immunology, have put forward valuable suggestions to guide clinical practice. Although the methodological quality of EB-CPGs is higher than CB-CPGs in general, they all have deficiencies in the following aspects, including obtaining the views and preferences of the target population, considering benefits and risks when formulating recommendations, introducing a detailed update plan, and providing implementation strategy for the recommendations or methods for managing potential conflicts of interest, similar to Dagens', Luo', and Zhao' studies (3, 4, 6). In view of the above topics, there are some examples of good practice, for example, conducting interviews and group surveys to collect information on treatment evidence from frontline experts fighting the disease (34); inviting patients recovering from COVID-19 to get involved in the guideline development panel (45); critically assessing new studies where these supersede previous outdated recommendations (14); providing available recommendation summaries in user-friendly and multilayered formats for clinicians and patients through the MAGIC app (55) or the provision of consultation decision aids to facilitate shared decision-making (45); and using the GRADEpro guideline development tool online software to conduct evidence-based CPGs (56). The methodology for guideline development to deal with public health emergencies is still a challenge, and methods for their development which ensure the rigor, timeliness and implementability of recommendations is a problem to be further explored by methodologists.

Recommendations relevant to chemoprophylaxis, diagnosis, antiviral treatments, and discharge management of COVID-19 varied in the guidelines. Chemoprophylaxis may be beneficial to reduce COVID-19 spread, which is important when lacking specific vaccines due to the high social and economic costs caused by social distancing of entire populations and blockade of entire cities. This method has been applied to other respiratory viruses; for example, healthcare workers who were exposed to high risk groups fought against the Middle East respiratory syndrome coronavirus using lopinavir-ritonavir plus ribavirin in South Korea (57). Unfortunately, there are still no effective and verified drugs for COVID-19 prophylaxis in the guidelines. However, a retrospective cohort study on family members and health care workers who were exposed to patients diagnosed with SARS-CoV-2 suggested that Arbidol could reduce risk of infection with the disease in hospital and family settings (58). SARS-CoV 2 vaccines may be beneficial for the prevention of COVID-19. The effectiveness and safety of them are still continuously ongoing trials. For instance, estimated BNT162b2 and mRNA-1273 COVID-19 vaccines effectiveness for prevention of infection was 90% for full immunization and 80% for partial immunization (59). Most commonly reported adverse effects of COVID-19 mRNA-1273 vaccine were localized pain, generalized weakness, headache, and myalgia (60). New evidence may inform decision making on chemoprophylaxis for healthcare personnel by policy makers in the future.

Diagnostic criteria for COVID-19 were not identical across the guidelines. What is more consistent is confirmation of diagnosis by testing positive for SARS-CoV-2 by real-time PCR. The main differences are the inclusion of other features, such as epidemiological history, serological tests, and clinical manifestations, as one of the bases for the diagnosis. Early studies have confirmed that 49–66% patients had contact with personnel in outbreak area (61). Up to now, asymptomatic infection of SARS-CoV-2 has become a worldwide concern. A recent study indicated that these cases may account for 60% of all infections and may trigger new outbreaks (62). Asymptomatic cases were significantly younger than those with symptomatic patients, had similar common incidence rate, and were more likely to come from high altitude and low mobility areas, with better history of epidemiology (63). Careful examination of the epidemiological history would help to identify asymptomatic patients that may have delayed symptoms after diagnosis. In addition, stability issues of RT-PCR testing of COVID-19 for hospitalized patients clinically diagnosed with SARS-CoV-2 are a problem. Li et al. reported a potentially high false negative rate of RT-PCR where results from several tests from the same patients at different points were inconsistent during the course of their diagnosis and treatment (64). Current systematic reviews have confirmed that the detection of anti-SARS-CoV-2 IgG and IgM had high diagnostic efficiency (2,282 patients with SARS-CoV-2 and 1,485 healthy persons or patients without SARS-CoV-2) (65) and a high sensitivity of chest CT for the detection of COVID-19 in regions with severe (3,186 patients) (66). The presentation of COVID-19 symptoms (such as fever, cough, myalgia/fatigue, leukocyte, and neutrophil counts) might be regarded as a surrogate marker for the disease' presence and severity (67, 68). Therefore, serological criteria, epidemiological history, clinical manifestations, and chest x-ray/CT should also be used for to assist diagnosis for COVID-19 infection during the current epidemic, counteracting possible false negative RT-PCR results if available.

Studies published after the deadline for analysis have been included here. Although there were no consistent recommendations on the usage of antiviral drugs, it does offer a few valuable suggestions, including antiviral drugs, such as hydroxychloroquine and remdesivir, for COVID-19. The majority of EB-CPGs did not recommend hydroxychloroquine +/– azithromycin to treat patients with COVID-19 because higher certainty benefits (e.g., mortality reduction) are now highly unlikely even if additional high quality randomized controlled trials would become available (14, 15, 18, 20, 21, 40, 45). Remdesivir is an antiviral drug with potent *in vitro* activity against a range of RNA viruses including MERS-CoV, SARS-CoV, there may be a favorable risk-benefit profile for remdesivir compared with no antiviral treatment in severe COVID-19 infection with limited safety data currently available for the drug (14–16, 20, 21, 24, 25, 34, 37, 47). In addition, Traditional Chinese medicine treatment may be beneficial for the treatment of COVID-19, including Lianhua Qingwen granules/capsules and Huashi Baidu granules. More new evidence concentrating on antiviral therapy continuously emerges. For example, early application of lopinavir / ritonavir+interferon-α can reduce the shedding time of sars-cov-2 (69); Early initiation with interferon) β- 1b, lopinavir, ribavirin combination therapy were more safe and effective than lopinavir alone in relieving symptoms, shortening length of stay in patients with mild to moderate COVID-19 (70); Lianhua Qingwen combined with Western medicine may have a significant effect and fewer side effects in the treatment of common patients with new coronavirus pneumonia (71). The new evidence above will help to update the recommendations of the guidelines.

The phenomenon that some discharge patients have tested positive for COVID-19 again after recovery has attracted a lot of attention. The included guidelines provided different suggestions on discharge criteria and precautions after discharge. As previously stated, Chest CT and X ray can be beneficial for COVID-19 diagnosis. Viral RNA was detected in 48.1% of patients' feces, even in the feces who have been diagnosed with negative results in respiratory tract samples (72). Thus, a nucleic acid test of upper airway specimens (nasopharyngeal and pharyngeal swabs) and fecal stool can be considered along with other criteria. Additionally, it may be necessary to continue isolation management and health status monitoring. A follow-up study for 651 patients recovered from COVID-19 revealed that 3% of the patients were positive for SARS-CoV-2 by RTqPCR in routine physical examination and the median time from discharge to retest with postive results was 15.0 days (73). Thus, the COVID-19 pandemic is a rapidly changing situation. The recommendations in the guidelines are also continuously changing. The evidence-based living guidelines are pursed (74, 75).

A strength of this review lies in the updated study (up to April 5, 2021) concentrating on hot topics, including chemoprophylaxis, diagnosis, antiviral therapy and discharge management of COVID-19 guidelines, at the same time and summarizing the recommendations. In addition, we defined the CPGs, distinguished EB-CPGs and CB-CPGs in order to gain the valuable recommendations developed by multidisciplinary experts and based on best evidence. However, there are several inevitable limitations in this current study. First, we did not compare the evidence and recommendation levels or different grade systems used in EB-CPGs. With new evidence emerging over time, some CPGs will be updated and evidence and recommendation levels may be changed or improved later. Second, we only searched the three medical databases and eight representative guidelines repositories, and some eligible EB-CPGs and CB-CPGs will thus have been missed.

## Conclusion

In general, the methodological quality of EB-CPGs is greater than CB-CPGs. But we still need to pay attention to gathering and synthesizing reliable the latest up-to-date information, involving the target population in guideline development and improving the implementability of the recommendations. As for the recommendations of COVID-19, SARS-CoV 2 vaccines are still going through ongoing trials; various diagnosis strategies, including serological criteria and CT for COVID-19, may be more effective if available; hydroxychloroquine +/– azithromycin may be not beneficial to treat patients with COVID-19, but remdesivir may be a favorable risk-benefit in severe COVID-19 infection; and isolation management and health status monitoring after discharge may be still necessary. Thus, chemoprophylaxis and antiviral drugs of COVID-19 still need more trials for confirmation.

## Data Availability Statement

The original contributions presented in the study are included in the article/Supplementary Material, further inquiries can be directed to the corresponding author/s.

## Author Contributions

Y-YW and X-TZ designed the study and formulated inclusion criteria. Y-YW, QS, HZ, QH, and B-HL searched and identified eligible guidelines. HZ, B-HL, M-ZL, and S-HH extracted important information. M-ZL and QS evaluated the quality of each included guideline using AGREE II. X-TZ and Y-HJ examined the data extraction forms. Y-YW, QH, and S-HH analyzed the data. Y-YW, X-TZ, XY, and Y-HJ contributed to discussed the findings. Y-YW, X-TZ, XY, and Y-HJ developed the final manuscript. All authors have read and approved the manuscript.

## Conflict of Interest

The authors declare that the research was conducted in the absence of any commercial or financial relationships that could be construed as a potential conflict of interest.
