# Supplementary material for: Quality of and Recommendations for Relevant Clinical Practice Guidelines for COVID-19 Management: A Systematic Review and Critical Appraisal
Source: Front Med (Lausanne). 2021 Jun 10;8:630765. doi: 10.3389/fmed.2021.630765 (PMC8248791; doi:10.3389/fmed.2021.630765)
Supplement: Supplementary file 1 [file Table_1.DOC]

**Supplementary TABLE 1** A list of the websites with COVID-19 guidelines.

| **Title** | **Websites** |
| --- | --- |
| World Health Organization (WHO) | <https://www.who.int/> |
| National Institute for Health and Care Excellence (NICE) | [https://www.nice.org.uk/guidance/](https://www.nice.org.uk/guidance/ng2) |
| Scottish Intercollegiate Guidelines Network (SIGN) | <https://www.sign.ac.uk/our-guidelines.html> |
| Guidelines International Network (GIN) | <https://www.g-i-n.net/library/international-guidelines-library/> |
| ECRI guideline trust | https://www.ecri.org/coronavirus-covid-19-outbreak-preparedness-center |
| BIGG international database of GRADE guidelines | <https://sites.bvsalud.org/bigg/en/biblio/> |
| Association of American Medical Colleges | <https://www.aamc.org/coronavirus-covid-19-clinical-guidance-repository> |
| National Institutes of Health (NIH) | https://www.nih.gov/coronavirus |
