# Supplementary material for: Quality of and Recommendations for Relevant Clinical Practice Guidelines for COVID-19 Management: A Systematic Review and Critical Appraisal
Source: Front Med (Lausanne). 2021 Jun 10;8:630765. doi: 10.3389/fmed.2021.630765 (PMC8248791; doi:10.3389/fmed.2021.630765)
Supplement: Supplementary file 3 [file Table_3.doc]

**Supplementary TABLE 3** Characteristic of included guidelines.

| **Guidelines title** | **Date of publication** | **Type of guideline** | **Publication country/region** | **Guideline developers** | **Topic** | **Funding** |
| --- | --- | --- | --- | --- | --- | --- |
| IDSA guidelines on the treatment and management of patients with COVID-19 (14) | April 5, 2021 | EB-CPG | the United States | Infectious Diseases Society of America | Diagnosis, antiviral therapy | The Centers for Disease Control and Prevention (grant number 6 NU50CK000477-04-01) |
| Australian guidelines for the clinical care of people with COVID-19 (15) | April 1, 2021 | EB-CPG | Australia | Australian National COVID-19 Clinical Evidence Task force | Chemoprophylaxis, antiviral therapy | the Australian Government  Department of Health, Victorian Government Department of Health and Human Services, the Ian Potter Foundation and the Walter  Cottman Endowment Fund |
| COVID-19 rapid guideline:  managing COVID-19(16) | March 23, 2021 | EB-CPG | the United Kingdom | National Institute for Health and Care Excellence | Antiviral therapy | Unclear |
| American College of Rheumatology guidance for COVID-19 vaccination  in patients with rheumatic and musculoskeletal diseases – Version 1(17) | March 17, 2021 | CB-CPG | the United States | American College of Rheumatology | Chemoprophylaxis | Supported by the American College of Rheumatology |
| Management of hospitalised adults with coronavirus disease-19 (COVID-19): a European Respiratory Society living guideline(18) | March 10, 2021 | EB-CPG | International cooperation | European  Respiratory Society, Chinese Thoracic Society | Antiviral therapy | Funded by the European Respiratory Society |
| WHO living guideline: drugs to prevent COVID-19(19) | March 2, 2021 | EB-CPG | International organization | World Health Organization | Chemoprophylaxis | None |
| Coronavirus disease 2019 (COVID-19)  treatment guidelines(20) | March 5, 2021 | EB-CPG | the United States | National Institutes of Health | Chemoprophylaxis, diagnosis, antiviral therapy | Unclear |
| Surviving Sepsis Campaign guidelines on the management of adults with coronavirus disease 2019 (COVID-19) in the ICU: first update(21) | March 1, 2021 | EB-CPG | International organization | Surviving sepsis campaign COVID-19 subcommittee | Diagnosis, antiviral therapy | Unclear |
| COVID-19 convalescent plasma: interim recommendations from the AABB(22) | February 14, 2021 | CB-CPG | the United States | American Association of Blood Banks | Antiviral therapy | Unclear |
| Multicenter interim guidance on use of antivirals for children with coronavirus disease 2019/severe acute respiratory syndrome coronavirus 2(23) | February 13, 2021 | CB-CPG | North American | Panel of pediatric infectious diseases physicians and pharmacists | Antiviral therapy | the Agency for Healthcare  Research and Quality (K12-HS026393 to K. C.) |
| 2021 update of the AGIHO guideline on evidence-based management of COVID-19 in patients with cancer regarding diagnostics, viral shedding, vaccination and therapy(24) | February 10, 2021 | EB-CPG | Germany | COVID-19 guideline panel of the Infectious Diseases Working Party of the German Society for Haematology  and Medical Oncology | Chemoprophylaxis, diagnosis, antiviral therapy | None |
| Should remdesivir be used for the treatment of patients with COVID-19? rapid, living practice points from the American College of Physicians (version 2)(25) | February 9, 2021 | EB-CPG | the United States | American College of Physicians | Antiviral therapy | the ACP operating budget |
| Clinical management of COVID-19 patients: living guidance(26) | January 25, 2021 | EB-CPG | International organization | World Health Organization | Diagnosis, antiviral therapy | Unclear |
| SARS-CoV-2 vaccination for patients with inflammatory bowel diseases: recommendations from an international consensus meeting(27) | January 20, 2021 | CB-CPG | International organization | the International Organization for  the Study of Inflammatory Bowel Disease | Chemoprophylaxis | None |
| Therapeutic strategies for severe COVID-19: a position paper from the Italian Society of Infectious and Tropical Diseases (SIMIT)(28) | January 18, 2021 | CB-CPG | Italy | Italian Society of Infectious and Tropical Diseases | Antiviral therapy | Unclear |
| Pragmatic recommendations for tracheostomy, discharge, and rehabilitation measures in hospitalized patients recovering from severe COVID-19 in low- and middle-income countries(29) | January 13, 2021 | EB-CPG | International cooperation | the COVID-LMIC Task Force and the  Mahidol-Oxford Research Unit | Discharge | the Wellcome Trust of Great Britain |
| Clinical practice guideline: recommendations on inpatient treatment of patients with COVID-19(30) | January 11, 2021 | EB-CPG | Germany | the Association of the  Scientific Medical Societies in Germany | Diagnosis | the German Robert Koch  Institute |
| Pragmatic recommendations for identification and triage of patients with COVID-19 disease in low- and middle-income countries(31) | January 6, 2021 | EB-CPG | International cooperation | the COVID-LMIC Task Force and the  Mahidol-Oxford Research Unit | Identification and triage of patients with COVID-19 | the Wellcome Trust of Great Britain |
| Clinical management of coronavirus disease 2019  (COVID-19) in pregnancy: recommendations of  WAPM-World Association of Perinatal Medicine(32) | November 26, 2020 | CB-CPG | International organization | World Association of Perinatal Medicine | Diagnosis, antiviral therapy | None |
| Algorithms for testing COVID-19 focused on use of RT-PCR and  high-affinity serological testing: a consensus statement from a  panel of Latin American experts(33) | November 21, 2020 | CB-CPG | Latin American | A panel of Latin American experts | Diagnosis | Roche Diagnostics |
| Chemoprophylaxis, diagnosis, treatments, and discharge management of COVID-19: an evidence-based clinical practice guideline (updated version)(34) | September 4, 2020 | EB-CPG | China | Evidence-Based Medicine Chapter of China International Exchange and Promotive Association for Medical and Health Care and Chinese Research Hospital Association | Chemoprophylaxis, diagnosis, antiviral therapy,  and discharge | the National Key Research and  Development Program of China (2020YFC0845500), the Special Project for  Emergency of Hubei Province (2020FCA008), and the First Level Funding of  the Second Medical Leading Talent Project in Hubei Province |
| COVID-19: interim guidance on  rehabilitation in the hospital and  post-hospital phase from a European  Respiratory Society- and American Thoracic  Society-coordinated international task force(35) | August 13, 2020 | CB-CPG | International cooperation | An ad hoc international task force including the European Respiratory Society and American Thoracic Society | Discharge | Unclear |
| Use of chest imaging in the diagnosis and management of COVID-19: a WHO rapid advice guide(36) | July 30, 2020 | EB-CPG | International organization | World Health Organization | Diagnosis, discharge | Government of Japan |
| Remdesivir for severe covid-19: a clinical practice guideline (37) | July 30, 2020 | EB-CPG | International cooperation | Multidisciplinary experts | Antiviral therapy | Unclear |
| Updated guidance on the management of  COVID-19: from an American Thoracic  Society/European Respiratory Society  coordinated International Task Force(38) | July 29, 2020 | CB-CPG | International cooperation | European Respiratory Society- and American Thoracic Society-coordinated international task force | Antiviral therapy | Unclear |
| Traditional Chinese medicine guidelines for coronavirus disease 2019(39) | July 18, 2020 | EB-CPG | China | Panel of Chinese experts | Antiviral therapy | Unclear |
| Guidelines for the pharmacological treatment of  COVID-19 (40) | July 13, 2020 | EB-CPG | Brazil | The task-force/consensus guideline of  the Brazilian Association of Intensive Care Medicine,  the Brazilian Society of Infectious Diseases and the  Brazilian Society of Pulmonology and Tisiology | Antiviral therapy | None |
| A consensus guideline of herbal medicine for coronavirus disease 2019(41) | July 5, 2020 | CB-CPG | Korea | 11 experts from the Society of Korean Medical Pulmonary Diseases | Chemoprophylaxis, antiviral therapy | the Traditional Korean Medicine R&D program funded by the Ministry of Health & Welfarethrough the Korea Health Industry Development Institute (KHIDI)(HB16C0006) |
| Rapid advice guidelines for management of children with COVID-19 (42) | May 22, 2020 | EB-CPG | China | 67 members of the working group came from 11 countries | Diagnosis, antiviral therapy | National Clinical Research Center for Child Health and Disorders (Children’ s Hospital of Chongqing Medical University, Chongqing, China) (NCRCCHD-2020-EP-01); Special Fund for Key Research and Development Projects in Gansu Province in 2020; The fourth batch of “Special Project of Science and Technology for Emergency Response to COVID-19” of Chongqing Science and Technology Bureau; Special funding for prevention and control of emergency of COVID-19 from Key Laboratory of Evidence Based Medicine and Knowledge Translation of Gansu Province (GSEBMKT-2020YJ01); The Fundamental Research Funds for the Central Universities (lzujbky-2020-sp14); Newton international fellowship from The Academy of Medical Science (NIF004/1012); UK National Institute of Health Research GOSH Biomedical Research Centre |
| Expert consensus for managing pregnant women and neonates born to mothers  with suspected or confirmed novel coronavirus (COVID-19) infection (43) | May 20, 2020 | EB-CPG | China | Multidisciplinary experts | Diagnosis | Unclear |
| Canadian society of thoracic radiology/  Canadian association of radiologists  consensus statement regarding chest  imaging in suspected and confirmed COVID-19 (44) | May 8, 2020 | CB-CPG | Canada | Canadian Society of Thoracic Radiology/  Canadian Association of Radiologists | Diagnosis | None |
| Treatment of patients with nonsevere and  severe coronavirus disease 2019: an evidence based  guideline (45) | April 29, 2020 | EB-CPG | International cooperation | Multidisciplinary experts | Antiviral therapy | None |
| Updated diagnosis, treatment and prevention of COVID‑19 in children:  experts’ consensus statement (condensed version of the second  edition) (46) | April 24, 2020 | CB-CPG | China | China National Clinical Research Center for Respiratory Diseases · National Center for Children’s  Health, Beijing, China Group of Respirology, Chinese Pediatric Society, Chinese Medical Association · Chinese  Medical Doctor Association Committee on Respirology Pediatrics · China Medicine Education Association  Committee on Pediatrics · Chinese Research Hospital Association Committee on Pediatrics · China Nongovernment  Medical Institutions Association Committee on Pediatrics · China Association of Traditional Chinese  Medicine, Committee on Children’s Health and Medicine Research · China News of Drug Information Association,  Committee on Children’s Safety Medication · Global Pediatric Pulmonology Alliance | Diagnosis, antiviral therapy | None |
| Interim guidelines on antiviral therapy for COVID-19 (47) | April 23, 2020 | EB-CPG | Korea | the Korean Society of Infectious Diseases, the Korean Society for Antimicrobial Therapy, and the Korean Society of Pediatric Infectious Diseases | Antiviral therapy | None |
| Imaging of coronavirus disease 2019: a Chinese expert consensus statement (48) | April 8, 2020 | CB-CPG | China | Radiology  Committee on Infectious and Inflammatory Disease, Chinese  Research Hospital Association, Radiology of Infection Branch,  Chinese Society of Radiology, Committee on Radiology of Infection,  Radiologist Branch, Chinese Medical Doctor Association | Diagnosis | National Science Foundation of China  (No. 62041601, No.61936013, No.81771806, No.81930049). National  Science and Technology Planning Project (2020ZX10001013). |
| The role of chest imaging in patient management during the COVID-19 pandemic (49) | April 7, 2020 | CB-CPG | International organization | the Fleischner Society | Diagnosis | Unclear |
| Guideline for critical care of seriously ill adults patients with coronavirus (COVID-19) in the Americans (50) | April 3, 2020 | EB-CPG | the United State | Pan American Health organization, World Health Organization | Antiviral therapy | Unclear |
| Perinatal-neonatal  management of  COVID-19 infection (51) | March 26, 2020 | EB-CPG | India | Federation of Obstetric & Gynecological Societies of India  National Neonatology Forum, India  Indian Academy of Pediatrics | Diagnosis, antiviral therapy, discharge | Unclear |
| Chinese expert consensus on the perinatal and neonatal management for the prevention and control of the 2019 novel coronavirus infection (first edition) (52) | February 6, 2020 | CB-CPG | China | the Working Committee on Perinatal and Neonatal Management for the Prevention and Control of the 2019 Novel Coronavirus Infection | Diagnosis, discharge | Unclear |

EB-CPG: evidence-based clinical practice guidelines; CB-CPG: consensus-based clinical practice guideline.
