# Supplementary material for: Quality of and Recommendations for Relevant Clinical Practice Guidelines for COVID-19 Management: A Systematic Review and Critical Appraisal
Source: Front Med (Lausanne). 2021 Jun 10;8:630765. doi: 10.3389/fmed.2021.630765 (PMC8248791; doi:10.3389/fmed.2021.630765)
Supplement: Supplementary file 4 [file Table_4.doc]

**Supplementary TABLE 4** Analysis of the included CPGs according to AGREE II (%).

| **Guidelines title** | **Scope and purpose** | **Stakeholder involvement** | **Rigour of development** | **Clarity of presentation** | **Applicability** | **Editorial independence** |
| --- | --- | --- | --- | --- | --- | --- |
| IDSA guidelines on the treatment and management of patients with COVID-19(14) | 94.44 | 33.33 | 90.63 | 100.00 | 18.75 | 50.00 |
| Australian guidelines for the clinical care of people with COVID-19(15) | 91.67 | 63.89 | 64.58 | 86.11 | 41.67 | 83.33 |
| COVID-19 rapid guideline: managing COVID-19(16) | 83.33 | 72.22 | 48.96 | 75.00 | 35.42 | 100.00 |
| American College of Rheumatology guidance for COVID-19 vaccination in patients with rheumatic and musculoskeletal diseases – Version 1(17) | 72.22 | 33.33 | 46.88 | 50.00 | 25.00 | 20.83 |
| Management of hospitalised adults with coronavirus disease-19 (COVID-19): a European Respiratory Society living guideline(18) | 91.67 | 83.33 | 64.58 | 86.11 | 77.08 | 50.00 |
| WHO living guideline: drugs to prevent COVID-19(19) | 97.22 | 91.67 | 78.13 | 97.22 | 66.67 | 100.00 |
| Coronavirus disease 2019 (COVID-19) treatment guidelines(20) | 83.33 | 50.00 | 33.33 | 83.33 | 22.92 | 33.33 |
| Surviving Sepsis Campaign guidelines on the management of adults with coronavirus disease 2019 (COVID-19) in the ICU: first update(21) | 88.89 | 77.78 | 65.63 | 94.44 | 39.58 | 87.50 |
| COVID-19 convalescent plasma: interim recommendations from the AABB(22) | 69.44 | 55.56 | 26.04 | 52.78 | 10.42 | 50.00 |
| Multicenter interim guidance on use of antivirals for children with coronavirus disease 2019/severe acute respiratory syndrome coronavirus 2(23) | 66.67 | 30.56 | 22.92 | 44.44 | 8.33 | 50.00 |
| 2021 update of the AGIHO guideline on evidence-based management of COVID-19 in patients with cancer regarding diagnostics, viral shedding, vaccination and therapy(24) | 55.56 | 33.33 | 26.04 | 33.33 | 8.33 | 62.50 |
| Should remdesivir be used for the treatment of patients with COVID-19? rapid, living practice points from the American College of Physicians (version 2)(25) | 66.67 | 44.44 | 36.46 | 52.78 | 33.33 | 100.00 |
| Clinical management of COVID-19 patients: living guidance(26) | 97.22 | 75.00 | 84.38 | 91.67 | 62.50 | 50.00 |
| SARS-CoV-2 vaccination for patients with inflammatory bowel diseases: recommendations from an international consensus meeting(27) | 58.33 | 36.11 | 27.08 | 52.78 | 14.58 | 50.00 |
| Therapeutic strategies for severe COVID-19: a position paper from the Italian Society of Infectious and Tropical Diseases (SIMIT)(28) | 55.56 | 38.89 | 27.08 | 63.89 | 14.58 | 54.17 |
| Pragmatic recommendations for tracheostomy, discharge, and rehabilitation measures in hospitalized patients recovering from severe COVID-19 in low- and middle-income countries(29) | 61.11 | 38.89 | 37.50 | 55.56 | 41.67 | 100.00 |
| Clinical practice guideline: recommendations on inpatient treatment of patients with COVID-19(30) | 38.89 | 47.22 | 15.63 | 33.33 | 10.42 | 29.17 |
| Pragmatic recommendations for identification and triage of patients with COVID-19 disease in low- and middle-income countries(31) | 55.56 | 38.89 | 29.17 | 52.78 | 31.25 | 100.00 |
| Clinical management of coronavirus disease 2019 (COVID-19) in pregnancy: recommendations of WAPM-World Association of Perinatal Medicine(32) | 50.00 | 33.33 | 14.58 | 52.78 | 20.83 | 100.00 |
| Algorithms for testing COVID-19 focused on use of RT-PCR and high-affinity serological testing: a consensus statement from a panel of Latin American experts(33) | 52.78 | 33.33 | 16.67 | 50.00 | 12.50 | 37.50 |
| Chemoprophylaxis, diagnosis, treatments, and discharge management of COVID-19: an evidence-based clinical practice guideline (updated version)(34) | 83.33 | 55.56 | 84.38 | 88.89 | 66.67 | 100.00 |
| COVID-19: interim guidance on rehabilitation in the hospital and post-hospital phase from a European Respiratory Society- and American Thoracic Society-coordinated international task force(35) | 61.11 | 36.11 | 23.96 | 63.89 | 18.75 | 50.00 |
| Use of chest imaging in the diagnosis and management of COVID-19: a WHO rapid advice guide(36) | 83.33 | 94.44 | 73.96 | 75.00 | 79.17 | 54.17 |
| Remdesivir for severe covid-19: a clinical practice guideline(37) | 80.56 | 75.00 | 70.83 | 94.44 | 62.50 | 50.00 |
| Updated guidance on the management of COVID-19: from an American Thoracic Society/European Respiratory Society coordinated International Task Force(38) | 66.67 | 33.33 | 20.83 | 63.89 | 25.00 | 50.00 |
| Traditional Chinese medicine guidelines for coronavirus disease 2019(39) | 83.33 | 38.89 | 54.17 | 50.00 | 10.42 | 20.83 |
| Guidelines for the pharmacological treatment of COVID-19(40) | 83.33 | 63.89 | 71.88 | 69.44 | 43.75 | 79.17 |
| A consensus guideline of herbal medicine for coronavirus disease 2019(41) | 72.22 | 36.11 | 33.33 | 63.89 | 12.50 | 50.00 |
| Rapid advice guidelines for management of children with COVID-19(42) | 80.56 | 72.22 | 71.88 | 83.33 | 52.08 | 50.00 |
| Expert consensus for managing pregnant women and neonates born to mothers with suspected or confirmed novel coronavirus (COVID-19) infection(43) | 47.22 | 25.00 | 29.17 | 50.00 | 20.83 | 50.00 |
| Canadian Society of Thoracic Radiology/Canadian Association of Radiologists consensus statement regarding chest imaging in suspected and confirmed COVID-19(44) | 55.56 | 33.33 | 22.92 | 69.44 | 27.08 | 100.00 |
| Treatment of patients with nonsevere and severe coronavirus disease 2019: an evidence based guideline(45) | 80.56 | 83.33 | 70.83 | 86.11 | 83.33 | 87.50 |
| Updated diagnosis, treatment and prevention of COVID‑19 in children: Experts’ consensus statement (Condensed Version of the Second Edition)(46) | 50.00 | 36.11 | 16.67 | 47.22 | 25.00 | 100.00 |
| Interim guideline on antiviral therapy for COVID-19(47) | 80.56 | 47.22 | 65.63 | 66.67 | 27.08 | 100.00 |
| Imaging of coronavirus disease 2019: A Chinese expert consensus statement(48) | 52.78 | 30.56 | 12.50 | 50.00 | 37.50 | 100.00 |
| The role of chest imaging in patient management during the COVID-19 pandemic(49) | 72.22 | 44.44 | 18.75 | 61.11 | 35.42 | 8.33 |
| Guideline for critical care of seriously ill adults patients with coronavirus (COVID-19) in the Americans(50) | 77.78 | 38.89 | 25.00 | 52.78 | 20.83 | 45.83 |
| Perinatal-neonatal management of COVID-19 infection(51) | 80.56 | 69.44 | 19.79 | 69.44 | 62.50 | 0.00 |
| Chinese expert consensus on the perinatal and neonatal management for the prevention and control of the 2019 novel coronavirus infection (first edition)(52) | 50.00 | 36.11 | 14.58 | 50.00 | 22.92 | 50.00 |
| EB-CPGs, Median, interquartile range (25, 75%) | 81.94(75.00,84.72) | 59.72(38.89,75.00) | 64.58(32.29,71.88) | 75.00(52.78,86.81) | 40.63(22.40,62.50) | 58.33(50.00,100.00) |
| CB-CPGs, Median, interquartile range (25, 75%) | 58.33(52.78,68.06) | 36.11(33.33, 36.11) | 22.92(16.67,26.56) | 52.78(50.00,63.89) | 20.83(13.54,25.00) | 50.00(50.00,7 7.08) |
| All, Median, interquartile range (25, 75%) | 72.22(55.56,83.33) | 38.89(34.72,66.67) | 33.33(22.92,65.63) | 63.89(51.39,83.33) | 27.08(18.75,42.71) | 50.00(50.00,100.00) |

EB-CPGs: Evidence-based clinical practice guidelines; CB-CPGs: Consensus-based guidelines.
