# Supplementary figures and images for: Quality of and Recommendations for Relevant Clinical Practice Guidelines for COVID-19 Management: A Systematic Review and Critical Appraisal
Source: Front Med (Lausanne). 2021 Jun 10;8:630765. doi: 10.3389/fmed.2021.630765 (PMC8248791; doi:10.3389/fmed.2021.630765)

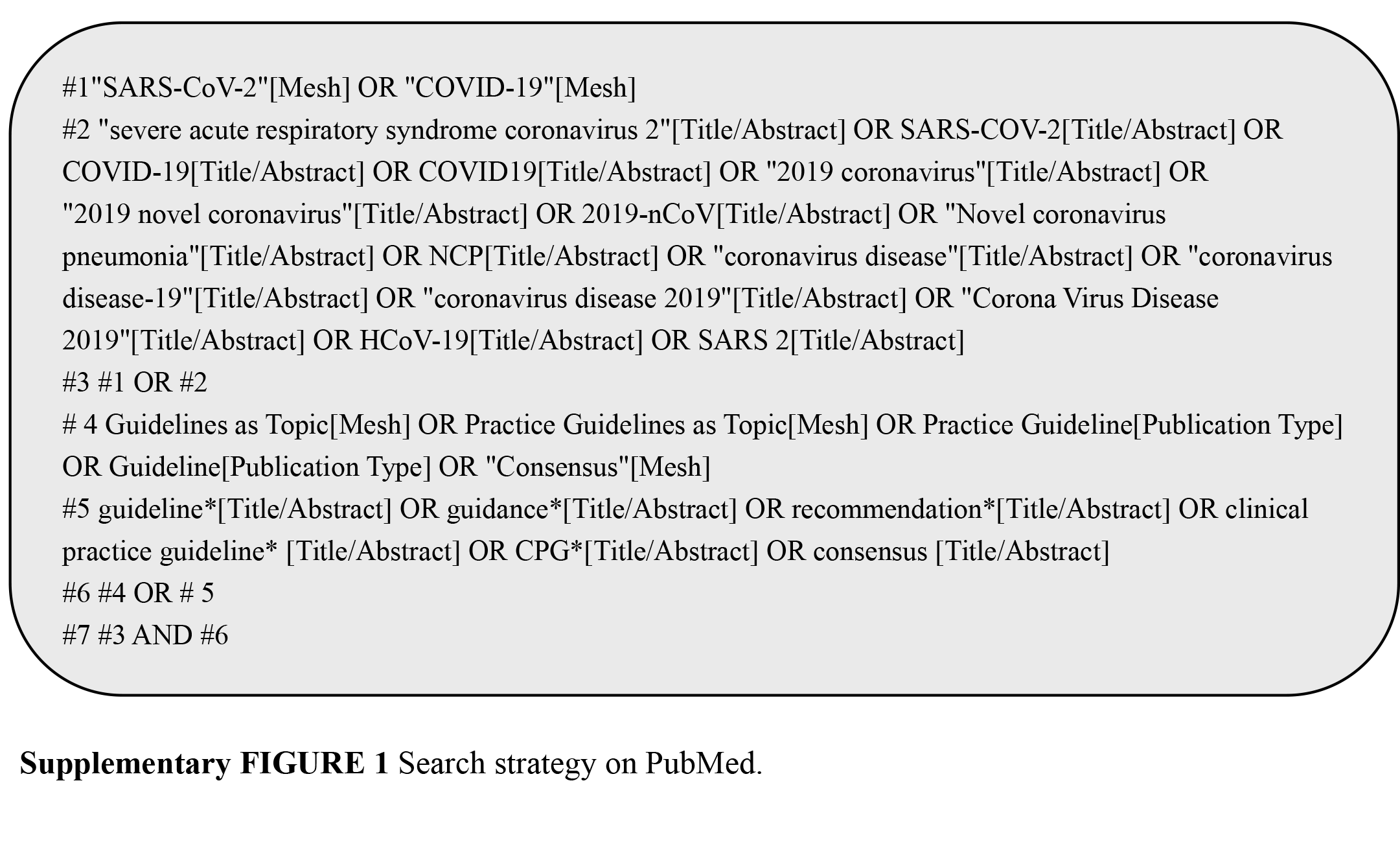

Supplement: Supplementary file 5 [file Image_1.TIF]
